# Do Anti-Angiogenic VEGF (VEGFxxxb) Isoforms Exist? A Cautionary Tale

**DOI:** 10.1371/journal.pone.0035231

**Published:** 2012-05-02

**Authors:** Sheila Harris, Madeleine Craze, Jillian Newton, Matthew Fisher, David T. Shima, Gillian M. Tozer, Chryso Kanthou

**Affiliations:** 1 Cancer Research United Kingdom Tumour Microcirculation Group, Department of Oncology, School of Medicine, University of Sheffield, Sheffield, United Kingdom; 2 Biomedical Centre, Owen Building, Sheffield Hallam University, Sheffield, United Kingdom; 3 University College London Institute of Ophthalmology, London, United Kingdom; Istituto Dermopatico dell’Immacolata, Italy

## Abstract

Splicing of the human vascular endothelial growth factor-A (VEGF-A) gene has been reported to generate angiogenic (VEGFxxx) and anti-angiogenic (VEGFxxxb) isoforms. Corresponding VEGFxxxb isoforms have also been reported in rat and mouse. We examined VEGFxxxb expression in mouse fibrosarcoma cell lines expressing all or individual VEGF isoforms (VEGF120, 164 or 188), grown *in vitro* and *in vivo*, and compared results with those from normal mouse and human tissues. Importantly, genetic construction of VEGF164 and VEGF188 expressing fibrosarcomas, in which exon 7 is fused to the conventional exon 8, precludes VEGFxxxb splicing from occurring. Thus, these two fibrosarcoma cell lines provided endogenous negative controls. Using RT-PCR we show that primers designed to simultaneously amplify VEGFxxx and VEGFxxxb isoforms amplified only VEGFxxx variants in both species. Moreover, only VEGFxxx species were generated when mouse podocytes were treated with TGFβ-1, a reported activator of VEGFxxxb splice selection in human podocytes. A VEGF164/120 heteroduplex species was identified as a PCR artefact, specifically in mouse. VEGFxxxb isoform-specific PCR did amplify putative VEGFxxxb species in mouse and human tissues, but unexpectedly also in VEGF188 and VEGF164 fibrosarcoma cells and tumours, where splicing to produce true VEGFxxxb isoforms cannot occur. Moreover, these products were only consistently generated using reverse primers spanning more than 5 bases across the 8b/7 or 8b/5 splice junctions. Primer annealing to VEGFxxx transcripts and amplification of exon 8b primer ‘tails’ explained the artefactual generation of VEGFxxxb products, since the same products were generated when the PCR reactions were performed with cDNA from VEGF164/VEGF188 ‘knock-in’ vectors used in the generation of single VEGF isoform-expressing transgenic mice from which the fibrosarcoma lines were developed. Collectively, our results highlight important pitfalls in data interpretation associated with detecting VEGFxxxb isoforms using current methods, and demonstrate that anti-angiogenic isoforms are not commonly expressed in mouse or human tissues.

## Introduction

**Figure 1:**
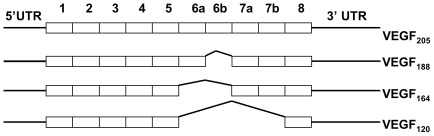
Murine VEGF-A splice variants. Several splice variants of VEGF are produced, by alternative splicing of exons 6 & 7. Exons 3 & 4 encode dimerisation and VEGF receptor binding sites, exon 7 encodes neuropilin binding sites and exons 6 & 7 encode heparin binding sites.

Vascular endothelial growth factor-A (hereafter referred to as VEGF) is a key regulator of angiogenesis, a process fundamental to the growth and metastasis of tumours [1], [2]. VEGF mRNA and protein are up-regulated in many cancers, with high VEGF levels often being associated with a poor prognosis [3]–[6]. Not surprisingly, many current vascular based anti-cancer therapies are targeted to inhibiting VEGF [7]–[10]. However, VEGF biology is complex. Both the human and murine VEGF genes are comprised of eight exons separated by seven introns [11], [12]. In man, at least nine different VEGF isoforms (VEGFxxx; where x denotes the number of amino acids) have been described due to alternative splicing of the VEGF gene, the most common and well studied being VEGF189, VEGF165 and VEGF121 (VEGF120, VEGF164 and VEGF188 in mouse) [13]–[15]. All VEGF isoforms contain exons 1 to 5 and 8 but differ in composition of remaining exons 6 to 7 (Fig. 1) and consequently binding to extracellular matrix and neuropilin co-receptors. VEGF165/164, which lack exon 6 but retain exon 7, represent the most abundantly expressed VEGF isoform in humans and mouse respectively [16]–[18]. The matrix binding characteristics of individual isoforms are thought to generate gradients *in vivo* that are important for angiogenesis [19]–[21]. While relatively little is known regarding the mechanistic functions of the different VEGF isoforms, *in vivo* studies using isoform-specific transgenic mice have revealed distinct roles in vascular growth, branching and patterning during development [22]–[24]. More recently, we demonstrated thattransgenic isoform-specific mouse embryos (VEGF-188, 164 or 120) gave rise to tumours with distinct morphological and functional vascular characteristics [25], mirroring some of the effects observed during development.

In 2002, a novel family of human VEGF-A splice variants was described (VEGFxxxb), formed by alternative splicing downstream of the conventional exon 8 within what was thought to be the 3′ untranslated region (3′ UTR) of the VEGF gene, resulting in the new open reading frame exon 8b [26]. VEGF165b has been described in normal human tissues, and shown to exhibit anti-angiogenic activity *in vitro* [26]–[33] and *in vivo* [26]–[30], [32], [34]–[37]. VEGFxxxb variants have been reported to account for up to 50% of total VEGF in some normal human tissues, whilst being down-regulated in cancer [15], [27]–[31], [38], [39]. Consequently, these observations led to the suggestion that it is the balance between the two families of VEGF that ultimately determines angiogenic outcome [15]. As current anti-VEGF strategies could potentially target both the pro- and anti-angiogenic forms of VEGF [40], this raises important questions for cancer therapies. However, there have been few studies reporting VEGFxxxb isoform expression in normal and tumour tissues since the initial discovery of VEGF165b [26]. While anti-angiogenic VEGF165b and more recently VEGF189b [41] and VEGF121b [42] have been described in man, the expression of these anti-angiogenic variants has only been described in the mouse at the protein level, using detection with an antibody that was initially developed for human use [43]. Therefore it is important to determine whether the mouse effectively models the human in this regard at both gene and protein levels. Alignment of the murine and human VEGF-A genes reveals conservation (86%) of the exon 8b sequence in the murine VEGF-A gene, predicting the possibility of a VEGFxxxb splicing reaction in mice. Interestingly, this would result in an exon 8b encoding seven amino acids (PLTGKTD) compared to the human exon8b, which encodes six amino acids (SLTRKD).

Here, we use reverse transcriptase PCR (RT-PCR) and a range of primer designs to dissect VEGF isoform expression in mouse and human cells and tissues, including tumours. In particular, we emphasise pitfalls associated with interpretation of PCR results. The genetic construction approaches used for the development of the VEGF isoform-specific transgenic mice, from which our fibrosarcoma cell lines were developed [25], makes the fibrosarcoma cells particularly useful for our studies. Specifically, *VEGFa^120/120^* mice were constructed by site-specific removal of exons 6 & 7, retaining VEGF intronic sequences, and as such these mice retain the potential to splice into exon 8b to create a putative VEGF120b isoform [22]. In contrast, the *VEGFa^164/164^* and *VEGFa^188/188^* mice were constructed using cDNAs encoding exons 4, 5, 7 & 8 (for VEGF164) or 4 to 8 (for VEGF188) [23]. Since in these mice exon 7 is fused to the conventional exon 8, expression of VEGF164b or VEGF188b respectively is excluded. Thus, splicing to generate VEGFxxxb variants could potentially occur in fibrosarcomas expressing exclusively VEGF120 (or wild type controls) but not in those expressing only VEGF164 or VEGF188 and hence these two fibrosarcoma lines provided endogenous negative controls.

## Materials and Methods

### Cell Culture and Cell Lines

The VEGF-specific fibrosarcoma cell lines, developed as described previously [25], were routinely maintained in high glucose Dulbecco’s modified Eagle’s medium (DMEM) supplemented with 10% foetal calf serum (FCS), 4 mM L-glutamine, puromycin (2 µg/ml) and geneticin (600 µg/ml). For the purposes of RNA extraction, cells were passaged at a ratio of 1∶6, 24 hr prior to harvesting to stimulate gene expression. Proliferating conditionally immortalised mouse podocytes (PCIPs) were kindly donated by Dr Jochen Reiser, University of Miami, USA. The cells were established from the Immortomouse, which carries a thermosensitive variant of the SV40 transgene. At the permissive temperature of 33°C, T-antigen expression can be stimulated by mouse γ-interferon (γ-INF), allowing rapid cell proliferation. Cells were routinely maintained in RPMI 1640 medium supplemented with 10% FCS, 4 mM L-glutamine as described previously [44]. For treatment with growth factors, cells were grown in six well plates (1×10^5^ cells per well) until 95% confluent. After starving for 24 hours in serum free medium, cells were treated with either 100 nM Insulin growth factor 1 (IGF-1) or 1 nM Transforming growth factor beta 1 (TGFβ-1) for 12 hours and processed for RNA/cDNA extraction as described below. Human embryonic kidney cells (HEK293) were obtained from the ATCC (CRL-1573) and maintained in Eagle’s Minimum Essential medium (EMEM) supplemented with 10% FCS and 4 mM L-glutamine. For the purposes of RNA extraction, cells were split at a ratio of 1∶2 to stimulate gene expression.

Generation of murine subcutaneous tumours and mouse and human tissue extraction.

All animal experiments were conducted according to United Kingdom Animals (Scientific Procedures) Act 1986 (UK Home Office Project Licence PPL40/3110) and with local University of Sheffield ethical approval. VEGF-specific fibrosarcomas were generated *in vivo* as we described previously [25]. Briefly, a cell suspension (1×10^6^ cells in 50 µl) was injected into the rear dorsum of 8–12 week old female severe combined immunodeficiency (SCID) mice and tumours were excised when they reached ∼8 mm mean diameter in size. Tumours were cut into smaller pieces and suspended in RNA later (Ambion) overnight at 4°C. The next day, RNA later was removed and tissues were stored at –80°C or processed immediately for RNA extraction. Mouse heart, lung liver and kidney organs, excised from non-tumour-bearing age-matched female SCID and CBA mice (5 of each), and a surgical specimen of human kidney tissue, obtained following ethics approval granted by the Sheffield Research Ethics Committee (Reference 06/Q2305/27) and patient written consent, were also processed as above.

### cDNA Synthesis and RT-PCR

**Figure 2:**
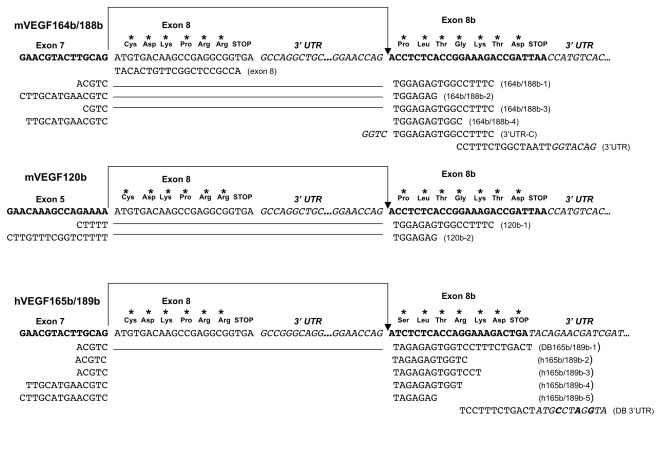
C-terminal exon sequences of murine and human VEGF-A genes. Top two panels show the predicted splicing reactions for murine VEGF188b, 164b and 120b (sequence in bold and arrowed), highlighting the exon 7/exon 8b and exon 5/exon 8b splice junction sequences. These splicing reactions would generate putative VEGFxxxb protein isoforms with a ***PLTGKTD*** C-terminus. The reverse PCR primers designed for this study are indicated below the exon sequences. The lower panel shows the corresponding sequence of the human VEGF-A gene C-terminus highlighting the exon 7/exon 8b splice junction that generates VEGFxxxb isoforms with a ***SLTRKD*** C-terminus. The reverse PCR primers used in the amplification reactions are indicated below the exon sequences and DB 165b/188b-1 and DB 3'UTR are as previously published [26]. The bases highlighted in bold exhibit mismatches from the published human VEGF-A sequence.

Total RNA was extracted from cultured cells (fibrosarcoma cells, HEK 293 cells; mouse podocytes) and converted to cDNA using the Cells to cDNA II kit (Ambion). Total RNA from tumours, mouse organs and human kidney tissue was extracted using the Mirvana^TM^ PARIS^TM^ kit (Ambion) and converted to cDNA using the Retroscript kit (Ambion) as per the manufacturer’s instructions. Briefly, 70–100 mg of tissue was homogenised in 500–600 µl of non-ionic detergent buffer supplied in the kit. Purified RNA was examined by denaturing formaldehyde gel electrophoresis to assess integrity. Prior to cDNA conversion, traces of genomic DNA were removed by treating 10 µg of total RNA with DNAse1 (Turbo DNA-free kit, Ambion). A portion of the RNA (1.5–2 µg) was then converted to cDNA by oligo-dT primed reverse transcription. Similar reactions were set up without reverse transcriptase (-RT) as controls to ensure absence of genomic DNA contamination. The cDNA (10% of total) was then routinely amplified in RT-PCR reactions using a number of different primer pairs. *General RT-PCR (mouse)*: Sequences of all murine (forward/reverse) primers are shown in Table 1 and reverse primers highlighted specifically in Fig. 2. When using primers that could amplify only pro-angiogenic VEGFxxx isoforms (exon3/exon8 and exon 3/3′ UTR-C) or both VEGFxxx and VEGFxxxb isoforms simultaneously (exon3/3′UTR and exon7a/3′UTR), RT-PCR was performed in 50 µl reactions comprising of 0.5 µM primers, 1.2 mM MgCl_2_, 200 µM dNTPs and 1 unit of Taq polymerase (2 × master mix supplied by Thermo scientific). Following an initial denaturation step at 94°C for 2 min, reactions were cycled 35 times, denaturing at 94°C for 1 min, annealing at 55°C for 1 min, and extending at 72°C for 1 min, followed by a final extension step of 72°C for 5 min. Approximately 4–20% of each PCR reaction was examined by gel electrophoresis using 2.5% agarose gels containing 0.5 µg/ml ethidium bromide. *Exon-specific RT-PCR (mouse)*: When performing isoform-specific RT-PCR to preferentially amplify VEGFxxxb isoforms, the same exon 3 or exon 7a forward primers but different isoform-specific reverse primers were used (see Table 1 & Fig 2). For detection of VEGF188b and VEGF164b, four different reverse primers were designed spanning different amounts of exon 7 and exon 8b (164b/188b-1, 164b/188b-2, 164b/188b-3 and 164b/188b-4). To detect VEGF120b, two different reverse primers were designed spanning different amounts of exon5 and exon 8b (120b-1 and 120b-2). RT-PCR reactions were performed routinely using conditions previously described to detect human VEGF165b [26]. Briefly, this involved an initial denaturation step at 94°C for 5 min, reactions were cycled 35 times, denaturing at 94°C for 0.5 min, annealing at 55°C or 63°C for 0.5 min, and extending at 72°C for 1 min, followed by a final extension step of 72°C for 2 min. The same conditions described for general RT-PCR (mouse) above were also employed to amplify VEGF isoforms from ‘knock-in’ DNA plasmid vectors (kindly provided by Dr Patricia D’Amoré) used in the generation of the VEGF single isoform expressing transgenic mice as templates [23]. Typically 100 ng of template DNA was used in PCR reactions. *RT-PCR using human cell and tissue cDNAs*: Sequences of all human forward/reverse primers are shown in Table 1 and reverse primers highlighted in Fig 2. RT-PCR was performed using two independent commercial sources of human cDNA extracted from cerebral cortex, bladder and kidney (AMS Biotechnology Ltd) and kidney, prostate and colon (Primer Design Ltd) as well as cDNA extracted in house from surgical patient normal kidney tissue. RT-PCR was also performed using HEK293 cDNA alongside mouse podocyte cDNA. PCR reactions were routinely performed in 25 µl using 1 µl of cDNA (AMS cDNAs) and 20 µl using 5 µl of cDNA (Primer Design cDNAs). Following an initial denaturation step at 95°C for 2 min, reactions were cycled 35 times, denaturing at 95°C for 1 min, annealing at 55°C or 63°C for 1 min, and extending at 72°C for 1 min, followed by a final extension step of 72°C for 5 min. To simultaneously amplify VEGFxxx and VEGFxxxb isoforms, the forward primers: DB exon4 or DB exon7a were used together with the reverse primer, DB 3′UTR (Fig. 2). To amplify only VEGFxxxb products, the forward primer: DB exon4 was used together with reverse primers DB165b/189b-1, h165b/189b-2, h165b/189b-3, h165b/189b-4 or h165b/189b-5. Only results of PCR reactions performed at 55°C are shown in the manuscript.

**Table 1:** Mouse and human PCR primer sequences.

Mouse Primers		
Mouse (Forward)		Tm
exon3	CAGGCTGCTGTAACGATGAA	60°C
exon7a	GTTTGTCCAAGATCCGCAGAC	64°C
exon7/8-C	GTTAAACGAACGTACTTGCAGATG	68°C
GAPDH-F	GCACAGTCAAGGCCGAGAAT	62°C
Mouse (Reverse)		
exon8	ACCGCCTCGGCTTGTCACAT	64°C
3′UTR-C	CTTTCCGGTGAGAGGTCTGG	64°C
3′UTR	GACATGGTTAATCGGTCTTTCC	64°C
3′UTR-1	GCATCCGAGTGAGGACATC	60°C
3′UTR-2	CCAGAAACAACCCTAATCTTCC	64°C
164b/188b-1	CTTTCCGGTGAGAGGT**CTGCA**	66°C
164b/188b-2	GAGAGGT**CTGCAAGTACGTTC**	64°C
164b/188b-3	CTTTCCGGTGAGAGGT**CTGC**	64°C
164b/188b-4	CGGTGAGAGGT**CTGCAAGTACGTT**	54°C
120b-1	CTTTCCGGTGAGAGGT**TTTTC**	62°C
120b-2	GAGAGGT**TTTTCTGGCTTTGTCC**	68°C
GAPDH-1	GCCTTCTCCATGGTGGTGAA	62°C
**Human Primers**		
Human (Forward)		
DB exon4	GAGATGAGCTTCCTACAGCAC	64°C
DB exon7a	GTAAGCTTGTACAAGATCCGCAGACG	78°C
Human (Reverse)		
DB165b/189b-1	TCAGTCTTTCCTGGTGAGAGAT**CTGCA**	80°C
h165b/189b-2	CTGGTGAGAGAT**CTGCA**	52°C
h165b/189b-3	TCCTGGTGAGAGAT**CTGCA**	58°C
h165b/189b-4	TGGTGAGAGAT**CTGCAAGTACGTT**	51°C
h165b/189b-5	GAGAGAT**CTGCAAGTACGTTC**	47°C
DB 3′UTR	ATGGATCCGTATCAGTCTTTCCT	66°C
DB exon8	TCACCGCCTCGGCTTGTCACAT	70°C

The sequences of all forward and reverse PCR primers used in this study are highlighted along with their corresponding melting temperatures. The junction spanning details of the VEGFxxxb isoform-specific primers are discussed in the main text (highlighted in bold above).

Efficient PCR conditions were confirmed by using primers designed to co-amplify GAPDH with VEGFxxxb in the same reactions. GAPDH products were readily amplified while transcripts corresponding to VEGFxxxb were undetectable probably due to preferential amplification of the more abundant GAPDH gene and depletion of reaction reagents in the system (data not shown).

### Sequencing of PCR Products

PCR products were excised from agarose gels under UV transillumination, and DNA extracted using the Quiaquick PCR purification Kit (Quiagen). The DNA was then sequenced using the exon 3 (for mouse PCR products) or exon 4 (for human PCR products) forward primers by fluorescent dideoxy termination sequencing (AB1370). Sequences were analysed by automated fluorescent chromatography and matched to the chromatograph manually. To verify the integrity of the murine VEGF 3′UTR exon 8b region in the fibrosarcomas RT-PCR analysis was performed using forward/reverse primers exon7/8-C and 3′UTR2 respectively (see Table 1). DNA Sequence analysis using the forward primer exon7a verified the integrity of the predicted exon 8b region and in addition, generated sequence data further downstream within the 3′UTR. Consistent with the different methods used in the generation of the VEGF^164^/VEGF^164^ & VEGF^188^/VEGF^188^ and VEGF^120^/VEGF^120^ mice [22], [23], an additional 85 bp sequence within the VEGF^164^/^188^ sequence, initiating at base 1141 (CCTCGAGGGACCTAATAACTTCGTATAGCATACATTATACGAAGTTATATTAAGGGTTATTGAATATGATCGGAATTGCTCGAGG), was detected which was not present in the VEGF^120^ sequence.

## Results

### Assessment of VEGF Isoform Expression by RT-PCR using Primers Designed to Amplify Both VEGFxxx and VEGFxxxb Isoforms

**Figure 3:**
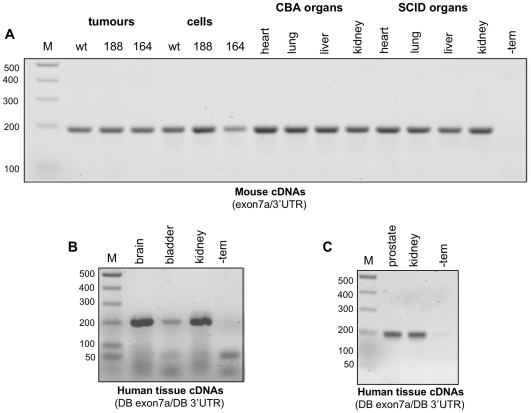
A general RT-PCR approach with primers to exon 7a and 3'UTR failed to detect VEGFxxxb isoforms in human and mouse cDNA extracts. RT-PCR reactions were performed using forward/reverse primers designed to amplify both VEGFxxx and VEGFxxxb isoforms from cDNAs extracted from mouse fibrosarcoma cell lines & tumours and normal mouse tissues (heart, lung, liver and kidney) as well as commercially sourced human tissue cDNAs. **A**, A single product corresponding to VEGF164/188 (194 bp) is evident in the panel of mouse cDNAs using forward and reverse primers designed to exon 7 and the 3'UTR (exon7/3′UTR) with no evidence of VEGF164b/188b (128 bp) products. **B** & **C**, A single product corresponding to VEGF165/189 (194 bp) is evident in human brain, bladder and kidney (AMS Biotechnology Ltd), and prostate and kidney (Primer Design Ltd) using forward and reverse primers designed to exon 7 (DB exon7a) and the 3'UTR (DB 3'UTR) respectively. No products corresponding to VEGF165b/189b (128 bp) are evident in any of these samples.

The existence of VEGFxxxb splice variants in human tissues has been reported in a number of studies using primarily reverse transcription (RT) PCR based approaches [14], [26]–[29], [38], [39], [41], [42], [45], [46]. To investigate expression of VEGF isoforms in our mouse fibrosarcoma cell lines, their corresponding solid tumour extracts and normal mouse and human cells/tissues, we adopted a PCR approach using primers designed to amplify VEGFxxx and VEGFxxxb families simultaneously, consistent with the primer design strategy previously reported for the identification of human VEGF165b [26]. cDNA extracts were prepared from mouse fibrosarcoma cells and tumours as well as mouse heart, lung, liver and kidney organs from two different strains of mice (CBA and SCID), to accommodate possible strain differences in VEGF isoform expression. In addition, two independent commercial sources of cDNA from human tissues (brain, bladder and kidney cDNAs from AMS Biotechnology Ltd, and kidney and prostate cDNAs from Primer Design Ltd) were included as positive controls. As shown in Fig. 3A–C, using forward and reverse primers designed to the murine or human exon7 and 3′UTR respectively, only one PCR product corresponding to VEGFxxx (194 bp) was consistently amplified from all the samples. This result held true for both independent commercial sources of human kidney cDNA, despite kidney being previously identified as an abundant source of VEGF165b [26], [31].

**Figure 4:**
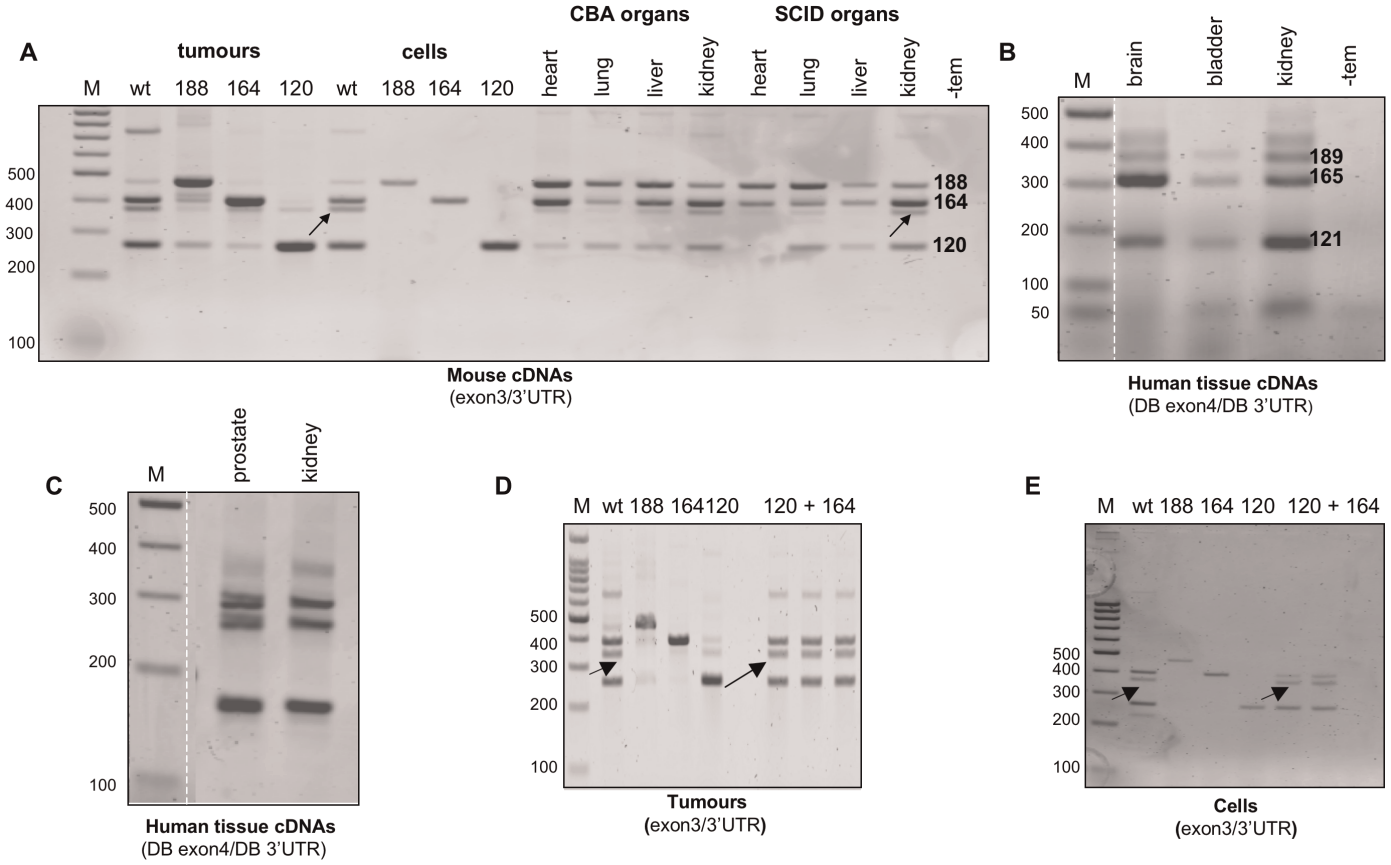
A general RT-PCR approach using primers to exon 3 or 4 and 3'UTR failed to detect VEGFxxxb isoforms in mouse and human cDNA extracts respectively. **A**, PCR products corresponding to VEGF188 (474 bp), VEGF164 (402 bp), VEGF164/120 heteroduplex (arrowed) and VEGF120 (270 bp) are evident in the panel of mouse cDNAs amplified using the 3′UTR reverse primer and a forward primer to exon 3. **B** & **C**, PCR products corresponding to VEGF189 (371 bp), VEGF165 (299 bp) and VEGF121 (167 bp) are evident in the commercial human tissue cDNAs amplified using the 3′UTR reverse primer (DB 3'UTR) and a forward primer to exon 4 (DB exon4). **D**, Amplification of the heteroduplex species (arrowed) a lso occurred when VEGF164 and VEGF120 tumour cDNAs were pooled and analysed by RT-PCR using exon3/3'UTR primers (lanes labelled 120+164). **E**, The same heteroduplex species was generated when cDNAs from VEGF164 and VEGF188 expressing fibrosarcoma cells were pooled and amplified as above (lanes labelled 120+164). M corresponds to a 100 bp ladder, whilst -tem represents a control PCR reaction in which water was used instead of cDNA template. The Figure contains data we published previously [25].

To validate these results and enable differentiation of all VEGF isoforms (ie. including VEGF120b), RT-PCR was performed on all samples using the same 3′UTR reverse primer(s) but this time an exon 3 forward primer (for mouse) and an exon 4 forward primer (for human). Again VEGFxxxb products would be expected to migrate approximately 66 bp below their VEGFxxx counterparts. As shown in Figure 4A, products consistent in size with VEGF188, 164 and 120 isoforms were detected in wt fibrosarcoma cell lines and tumours, while only the appropriate single isoform was evident in the isoform-specific cell lines and tumours, consistent with data we published previously [25]. Products corresponding to these isoforms were also detected in our panel of normal mouse tissues, albeit in differing abundances. Qualitative assessment of this data infers that VEGF188 appears the most abundant isoform in mouse lung and is also highly expressed in the heart, whilst VEGF164 appears the predominant isoform in mouse kidney, results consistent with the literature [47], [48]. Similarly, products corresponding to VEGF121, VEGF165 and VEGF189 were detected in our panel of human tissue cDNAs (Fig. 4B & C), however again no VEGFxxxb isoforms were detected, consistent with our previous mouse and human data (Fig. 3A–C). Interestingly, a smaller PCR product migrating just below VEGF164 (∼380 bp) was consistently observed in wt fibrosarcoma cell and tumour cDNAs [25] as well as cDNAs from normal mouse tissues, especially mouse kidney (see Fig. 4A, product denoted by arrows). Whilst this fragment was larger than expected for VEGF164b (323 bp), its presence, hinted to the possibility that at least for VEGF164, a sister murine VEGFxxxb isoform might exist. To determine the identity of this PCR product, high-resolution agarose gel electrophoresis was performed and the band was excised, purified and sequenced. The resultant chromatogram revealed a mixed sequence, whereby the sequence diverged at the end of exon 5, common to all VEGF isoforms, and then resolved to a single readable sequence again at the 3′ end. Analysis of this sequence showed that it resolved to a mixture of VEGF164 and VEGF120, suggesting that these two isoforms formed a heteroduplex. RT-PCR reactions performed using a mixture of cDNA templates from VEGF164 and VEGF120 tumours confirmed heteroduplex generation, since under these conditions a similar PCR product migrating just below the corresponding VEGF164 product was observed (Fig. 4D). DNA sequencing of this product revealed the same heteroduplex sequence described above (data not shown). To prove conclusively that this product is a PCR artefact and not a VEGFxxxb product derived from circulating mouse cells that infiltrated the tumours, the same proof of principle PCR was also performed using a mixture of cDNA templates isolated from the fibrosarcoma VEGF164 and VEGF188 cell lines. As shown in Figure 4E the same heteroduplex product was observed, thus confirming that VEGF164/120 can form a heteroduplex in RT-PCR reactions. Moreover, this confirmed that this transcript did not represent VEGFxxxb.

**Figure 5:**
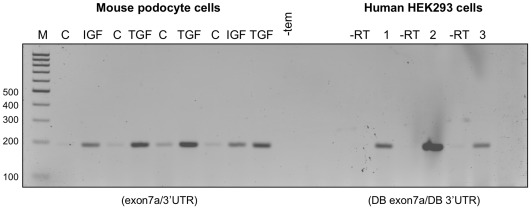
Effect of IGF-1 and TGFβ-1 on expression of VEGF isoforms in mouse podocytes. Cell lysates were prepared from untreated control (C) podocyte cells or cells treated with either 100 nM IGF-1 (IGF) or 1 nM TGFβ-1 (TGF) for 12 hours in serum free media. Results from three independent experiments are shown. RT-PCR using general primers designed to simultaneously amplify both VEGFxxx and VEGFxxxb isoforms (exon7a/3'UTR) revealed only VEGFxxx (194 bp). Qualitative assessment of the PCR products suggests that treatment with either growth factor increased VEGFxxx expression. The same RT-PCR strategy using three different extracts (1, 2, 3) from HEK 293 cells similarly revealed only VEGFxxx (194 bp). (-tem) corresponds to reactions containing water instead of cDNA. (-RT) corresponds to reactions containing water instead of reverse transcriptase.

To address more conclusively VEGFxxxb expression in mouse, we next performed mechanistic studies using growth factors reported to influence VEGF splice site selection choice in human podocytes (PCIPs) [14], [32]. Murine podocytes were grown at the permissive temperature of 33°C and then treated with either IGF-1 (reported to favour proximal splice site selection in exon 8 and VEGFxxx expression in human PCIPs) or TGFβ-1 (reported to favour distal splice site selection in exon 8 and VEGFxxxb expression in human PCIPs). As shown in Figure 5, RT-PCR using the exon7a/3′UTR primers only amplified VEGFxxx isoforms, for which there was qualitatively increased expression with addition of either growth factor. Interestingly, the same RT-PCR strategy also only amplified VEGFxxx species from HEK293 cells previously reported to express both VEGFxxx and VEGFxxxb isoform families [32]. Three repeats of these experiments are highlighted in Figure 5.

### Assessment of VEGF Isoform Expression by VEGFxxxb Isoform Specific RT-PCR

**Figure 6:**
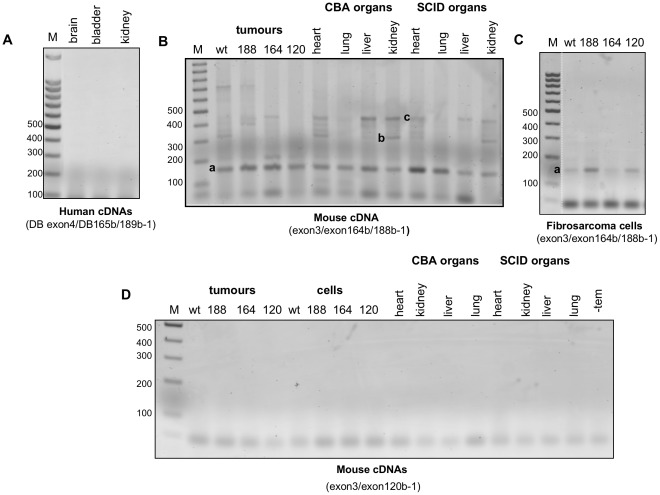
Characterisation of VEGF isoform expression in mouse and human cDNA extracts using VEGFxxxb isoform-specific RT-PCR. **A**, RT-PCR using an exon 4 forward primer (DB exon4) and an exon8 b/7 isoform-specific reverse primer (DB165b/189b-1) failed to detect any PCR products in commercial human tissue cDNAs (AMS Biotechnology Ltd.). **B** & **C**, RT-PCR using an exon 3 forward primer and a reverse primer designed to amplify VEGF164/188 (164b/188b-1) detected a single PCR product in mouse fibrosarcoma cell lines, as well as a number of PCR products in solid fibrosarcomas and normal CBA and SCID mouse tissues. Product a represents a truncated VEGF species containing exons 3, 4 & 8b. Product b represents a product identical in sequence to VEGF164b. Product c represents a product exhibiting 100% identity to murine acetyl co-A acetyl transferase. **D**, No products corresponding to VEGF120b were detected in mouse cDNAs when RT-PCR was performed using the same exon 3 forward primer but a reverse primer designed to amplify VEGF120b (120b-1).

Results described above suggested that VEGFxxxb isoforms were not expressed in the fibrosarcoma cells, tumours and normal mouse and human cell lines and tissues tested. To investigate this more stringently, discriminating isoform-specific RT-PCR was performed using the same forward primers as previously, but reverse primers designed to exclusively amplify murine VEGF164b and VEGF188b (exon3/164b/188b-1) and human VEGF165b and VEGF188b (DB exon4/DB165b/189b-1), which span the corresponding exon8b/exon7 splice junctions (see Fig. 2). As shown in Figure 6A, contrary to the literature, and consistent with our previous results, no VEGFxxxb products were detected in human cDNAs using a reverse primer spanning 5 bases complimentary in sequence to exon 7 (DB165b/189b-1). In contrast, several PCR products were detected in our panel of mouse cDNAs using a similar primer containing 5 bases complimentary in sequence to the murine exon 7 (Fig. 6B). DNA sequencing revealed that the abundant species, approximately 150 bp (Fig. 6B & C, product **a**) in all samples, represented a novel truncated VEGF transcript comprising exons 3, 4 and 8b, while the less abundant product, approximately 300 bp in wt tumours and heart and kidney mouse tissues (Fig. 6B, product **b**) corresponded to a putative VEGF164b transcript as confirmed by the presence of an exon7/exon8b junction sequence. The faint product migrating just above the 400 bp marker (Fig. 6B, product **c**) exhibited 100% identity to acetyl co-A acetyl transferase. However, of these products, only the truncated VEGF was reproducibly amplified in repeats of this experiment. Indeed this was the only species detected in the mouse fibrosarcoma cells (Fig. 6C) using this strategy. Repeating the strategy, with a murine VEGF120b specific reverse primer (120b-1) spanning 5 bases complimentary in sequence to exon 5 failed to reveal VEGF120b products in any mouse samples (Fig. 6D).

### Further Assessment of VEGFxxxb Isoform Expression in Mouse Fibrosarcoma Tumours Expressing All or Single VEGF Isoforms using a Refined Panel of VEGFxxxb Isoform-specific PCR Primers

**Figure 7:**
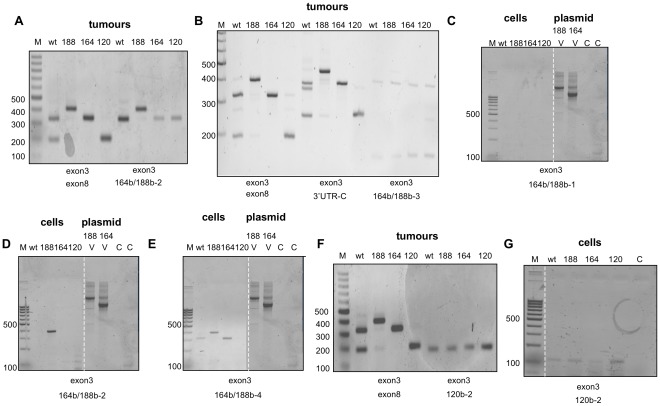
Discriminating VEGF isoform-specific RT-PCR highlights the importance of primer design in influencing detection of putative VEGFxxxb products. RT-PCR was performed using an exon 3 forward primer together with different VEGFxxxb isoform specific reverse primers designed to detect VEGF188b and/or VEGF164b or VEGF120b. **A**, RT-PCR using a reverse primer spanning 13 bases across the exon8b/exon7 junction (164b/188b-2) amplified a putative VEGF164b (311 bp) product in wt, VEGF164 & VEGF120-expressing tumours and a putative VEGF188b product (383 bp) in VEGF188-expressing tumours. **B**, More discriminating RT-PCR using a 164b/188b-3 reverse primer spanning only 4 bases across the exon8b/7 splice-junction failed to reveal VEGFxxxb PCR products. The ability to detect VEGFxxx products in reactions using exon8 and 3′UTR-C reverse primers highlights efficient PCR conditions. **C**, No products corresponding to VEGFxxxb isoforms were generated by RT-PCR using cDNA from mouse fibrosarcoma cells and a reverse primer spanning 5 bases across the exon8b/7 junction (164b/188b-1); VEGF188b (1209 bp) and VEGF164b (1137 bp) products were amplified from pPNT*VEGF^164^* (V^164^) & pPNT*VEGF^188^* (V^188^) ‘knock-in’ plasmid vector cDNA templates [23]. **D** & **E**, Putative VEGF188b and/or VEGF164b products were detected in RT-PCR reactions using reverse primers spanning 13 bases across the exon8b/7 junction (164b/188b-2 & 164b/188b-4) in fibrosarcoma cells and pPNT*VEGF^164^* & pPNT*VEGF^188^* plasmids. **F** & **G**, Products corresponding to VEGF120b (179 bp) species were readily detected in all fibrosarcoma tumour and cell extracts with a VEGF120-specific reverse primer spanning 16 bases across the exon8b/5 junction (120b-2), whilst no VEGF120b products were generated when the reverse primer spanned only 5 bases across the 8b/5 junction (120b-1).

**Figure 8:**
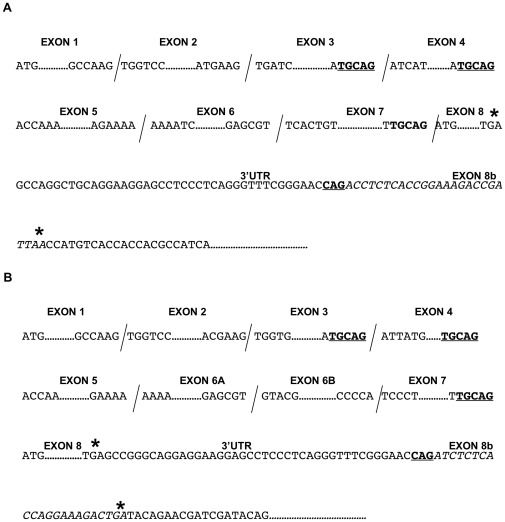
Exon splice junction sequences in the murine and human VEGF-A genes. Exon junction sequences are shown for the murine VEGF-A gene (**A**) and human VEGF-A gene (**B**), highlighting the sequence **TGCAG**, which exists at the end of exons 3, 4 & 7 (in-frame stop codons are asterisked). Since any VEGF164b/188b reverse primer sequence will contain sequence complementary to these bases, this highlights the possibility of obtaining truncated VEGF PCR products. The sequence **CAG** also occurs within the 3′UTR, immediately prior to the start of the predicted exon8b sequence (shown in italics), demonstrating the need for VEGF164b/18b isoform-specific primers to contain a minimum of four bases that span the exon8b/exon7 junction.

While the isoform-specific RT-PCR above did detect a putative VEGF164b product in wt fibrosarcoma tumours (as well as mouse heart and kidney), the irreproducibility associated with its detection made it difficult to formally determine whether it indeed represented a bonafide transcript. To address this more stringently, RT-PCR was performed next using cDNA extracted from fibrosarcoma tumours and a panel of refined isoform-specific primers, varying in the amount of sequence spanning the exon7/exon8b splice junction (see Fig. 2). For completeness, similar reactions were also performed in parallel using VEGFxxx isoform-specific primers, to ensure efficient PCR conditions. As shown in Fig. 7A, RT-PCR using primers designed to amplify VEGFxxx products (exon3/exon8) detected VEGF121, 164 and 188 in the tumour samples. Interestingly, when the same forward primer was used together with a reverse primer incorporating seven bases of exon 8b complementary sequence and fourteen bases of sequence complementary to exon7 (primer 164b/188b-2), products corresponding to VEGF164b (311 bp) were amplified in wt, 164 and 120 tumours, whilst a product corresponding to VEGF188b (383 bp) was amplified from VEGF188 tumours. Sequencing of all PCR products generated with the VEGFxxxb reverse primer confirmed presence of the exon7/exon8b junction sequence as defined by the primer design. The VEGF164b product detected in the VEGF120 fibrosarcomas could in principle reflect VEGF164b derived from the mouse host. However, when the RT-PCR was repeated using the same forward primer (exon3) but a reverse primer, which this time incorporated 16 bases of exon 8b complimentary sequence and only 4 bases of complimentary sequence to the end of exon7 (164b/188b-3), no VEGFxxxb products were obtained (Fig. 7B), even when higher stringency PCR conditions (63°C) were used (data not shown). Although very faint bands were evident in all four tumour types using this VEGF164/188b reverse primer (approximately 380 bp) these were not of the expected size for VEGFxxxb products (VEGF188b: 392 bp and VEGF164b: 320 bp) and furthermore were consistent in size across all four tumour lines, strongly suggesting that they were non-specific amplification artefacts (Fig. 7B). It should be noted that as defined by the VEGF-A gene sequence, the last 3 bases of exon 7 (^5′^CAG^3′^) are in common with the 3 bases prior to the start of exon8b within the 3′UTR, necessitating that at least 4 bases of sequence complementary to exon 7 have to be included before the primer becomes ‘isoform-specific’ (see Fig. 8). To demonstrate the isoform-specificity of these primers, a further control reverse primer (3′UTR-C) was also included which incorporated the same 16 bases of exon8b complementary sequence but 4 bases complementary to the 4 bases of murine VEGF-A 3′UTR sequence prior to the start of exon8b, and therefore did not span the VEGFxxxb/xxx splice junction. As such, this primer could amplify both VEGFxxx and VEGFxxxb isoforms. As shown in Figure 7B, products corresponding to VEGFxxx isoforms as well as the VEGF164/120 heteroduplex were observed using the control reverse primer, consistent with efficient PCR, and our previous data (Fig. 4D). However, no products corresponding to VEGFxxxb isoforms were detected. Together, these results suggested that the putative VEGFxxxb products observed are artefactual VEGFxxxb products obtained through mispriming of the reverse primer from existing VEGFxxx transcripts. To address this more conclusively, and to remove possible ambiguities associated with using tumour cDNA extracts, we next performed similar RT-PCR reactions as described above, but this time using cDNAs extracted from fibrosarcoma cells expressing either all (wt) or single VEGF isoforms. In parallel we also tested the same primer combinations on plasmid DNAs corresponding to the original ‘knock-in’ vectors used to create the VEGF164- and VEGF188-expressing transgenic mice. We argued that PCR using these purified plasmid DNAs would provide a definitive control to demonstrate the possibility that any putative VEGFxxxb products detected were indeed artefactual, since the nature of construction of these plasmids (the fact that in both cases exon 7 of VEGF cDNA was fused to exon 8), precludes VEGFxxxb splicing. Interestingly, as shown in Figs. 7 C, D & E, as the reverse primer design incorporated increasing complementarity to exon 7 across the exon8b/7 junction (from 5 bases across in Fig. 7C to 13 & 14 bases across in Fig. 7D & E respectively, see Table 1) putative VEGF164b and VEGF188b products were increasingly detected in the corresponding cell extracts. Most importantly however, abundant amplification products were observed for VEGF164b and VEGF188b using plasmid DNA as a template, thus proving conclusively that VEGFxxxb isoform specific reverse primers can amplify artefactual VEGFxxxb products from existing VEGFxxx templates. It should be noted that the products generated using the plasmid cDNAs differed in size from the corresponding products amplified from fibrosarcoma cDNAs. This is due to the design of the ‘knock-in’ vectors in which the VEGF cDNAs inserted into the pPNT backbone to create pPNT.*VEGF^164^* and pPNT.*VEGF^188^* respectively [23], retain the intron following exon3 (826 bp), so that the corresponding artefactual VEGF164b and VEGF188b amplified products would be 1137 bp (VEGF164b) and 1209 bp (VEGF188b) respectively. For completeness, and to provide further proof of principle, we next revisited our VEGF120 PCR data to determine whether manipulating the primer design around the exon8b/exon5 splice junction could influence the products detected. Interestingly, Figure 7F shows that a product (179 bp) was indeed now amplified in VEGF120 fibrosarcomas, using a reverse primer (120b-2) incorporating 16 bases of complementary sequence to exon 5, despite being undetected previously using a primer spanning only 5 bases of complementary sequence to exon 5 (120b-1). We believe this product represents a VEGF120 isoform, and the same product detected in the other isoform-specific fibrosarcomas, reflect VEGF120 derived from the mouse host, consistent with mispriming of the reverse primer from VEGFxxx species. Alternatively, since VEGF164 and VEGF188 contain exons 1–5, there is the possibility that this product was generated through annealing and mispriming of the reverse primer from exon5 in VEGF164 and VEGF188 species in these tumours rather than mouse host cells. This was confirmed when a similar result was obtained using cDNA templates extracted from the corresponding fibrosarcoma cell lines (see Fig. 7G). Collectively, these data strongly suggested that none of the fibrosarcoma tumours express VEGFxxxb isoforms.

**Figure 9:**
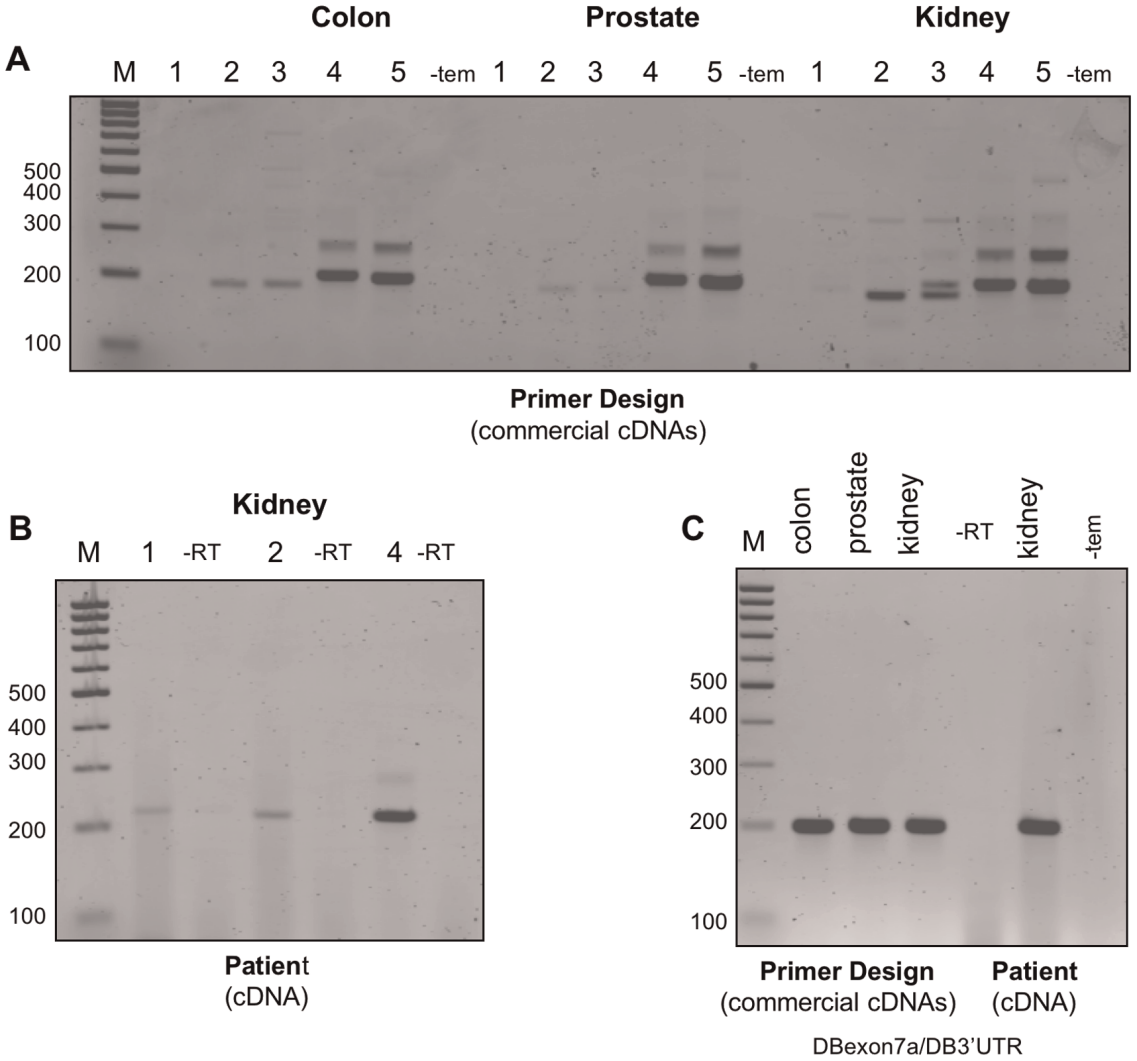
Discriminating VEGF isoform-specific RT-PCR using human cDNA extracts further highlights the importance of primer design. **A**, Human cDNAs from colon, prostate and kidney (Primer Design Ltd) were amplified in RT-PCR reactions using an exon 4 forward primer together with either DB165b/189b-1 (lane 1), h165b/189b-2 (lane 2), h165b/189b-3 (lane 3), h165b/189b-4 (lane 4) or h165b/189b-5 (lane 5). Primer sequences are highlighted in Table 1 & Fig 2. A putative VEGF165b (∼212 bp) product is evident in lanes 2 & 3, but absent in lane 1. All three reverse primers spanned 5 bases across the 8b/7 junction, however differed in their melting temperatures (see main text), with h165b/189b-2 & 3 exhibiting more compatible Tms with the forward primer. A similar 165b product was more abundantly amplified in lanes 4 & 5 using reverse primers spanning more bases (13 or 14 respectively) across the 8b/7 splice junction. In addition, these latter reverse primers also amplified a product corresponding to 188b (∼283 bp). However DNA sequencing revealed that the product amplified in lanes 2 & 3 was not VEGF but LIM domain only, protein isoform 7, confirming that detection of apparent VEGFxxxb species was only evident using 8b/7 reverse primers with increasing complementary sequence to exon7. **B**, Similar results were obtained in RT-PCR reactions using cDNA extracted from a normal kidney patient biopsy and amplified with the exon 4 forward primer and the DB165b/189b-1 (lane1), h165b/189b-2 (lane 2) and h165b/189b-4 (lane 4) reverse primers. **C**, RT-PCR using the DBexon7/DB3'UTR forward/reverse primers capable of simultaneously amplifying VEGFxxx & VEGFxxxb, confirmed only VEGFxxx (194 bp) amplification in the human samples tested.

It was surprising that, in our hands, the primer pair reported in the literature to amplify human VEGFxxxb isoforms failed to reveal VEGFxxxb transcripts in any of the human commercial cDNA extracts tested (DBexon4/DB165b/189b-1). However, closer examination of the melting temperatures (Tm) of these forward and reverse primers revealed that they differed in Tm by almost 20°C, conditions unfavourable for successful PCR amplification. To address this, we revisited the human VEGFxxxb isoform-specific primer design strategy and performed repeat reactions using the previous primer pair but this time including two additional isoform-specific reverse primers (h165b/189b-2 & h165b/189b-3) which spanned the same 5 bases across the 8b/7 junction but which had Tms similar to, and therefore more compatible with the forward primer (see Fig. 2). Interestingly, Figure 9A (lanes 2 & 3) shows that a putative VEGF165b product could now be detected in the commercial colon, prostate and kidney cDNAs (Primer Design). Moreover the apparent product appeared more abundantly amplified (lanes 4 & 5), when the reverse primer was manipulated to contain fewer bases complementary in sequence to exon 8b (either 7 or 11 bases, see Fig 2) and more bases complementary in sequence to the end of exon 7 (either 13 or 14, see Fig 2). An additional putative VEGF189b product was also evident when using these reverse primers (lanes 4 & 5, upper band). The same putative VEGF165b product was also obtained using cDNA extracted in house from normal human kidney (Fig. 9B). Since the PCR strategy using primers designed to simultaneously amplify both VEGFxxx and VEGFxxxb isoforms failed to amplify VEGFxxxb species from any of these samples (Fig 9C), this would argue that the products described above could represent misprimed VEGF165 and VEGF189 species (with an artefactual 8b ‘tail’) which increases in intensity when there are more complimentary bases to exon 7 promoting better annealing of the primers. Moreover, the fact that products differed in abundance (compare lanes 2 & 3 with 4 & 5) despite the fact that equivalent amounts of cDNA were used in the isoform-specific PCR reactions further supports this argument. It should be noted however that the putative VEGF165b products in Figure 9A, lanes 2 & 3 were slightly smaller than those in lanes 4 & 5. Sequencing revealed that indeed products in lanes 4 & 5 corresponded to VEGF165b, while products in lanes 2 & 3 showed 100% identity to an unrelated gene, namely LIM domain only protein 7 isoform. Hence, only when the VEGFxxxb reverse primer shows more complementarity to exon 7, across the exon8b/7 junction are putative products corresponding to VEGF165b generated, consistent with the data obtained in mouse using our refined panel of VEGFxxxb isoform-specific primers.

## Discussion

VEGF isoforms have always been considered as pro-angiogenic until recent reports described corresponding anti-angiogenic counterparts, formed by distal splicing of exon 8 of the VEGF gene. Using an RT-PCR based approach we were consistently unable to detect VEGFxxxb expression in mouse and human cells and tissues. Importantly, our results demonstrate that any VEGFxxxb-like transcripts that we did detect were most likely misprimed VEGFxxx amplification artefacts, highly dependent on PCR primer design. Recent literature addressing VEGF165b levels in normal human tissues by RT-PCR and ELISA-based approaches suggests that VEGFxxxb accounts for more than 50% of total VEGF in pancreatic islets, colon and vitreous humour, and a significant proportion in kidney lung and prostate [15]. However, our data questions this interpretation, since surprisingly, we did not detect VEGFxxxb in cDNAs extracted from tissues or cells previously reported to express VEGF165b abundantly [15], [26], [27], [49]. Analysis of human kidney cDNAs from two independent commercial sources as well as cDNA we prepared ‘in house’ from kidney and HEK293 cells confirmed this finding. Similarly, mouse podocytes only expressed VEGFxxx, even following stimulation with TGFβ-1 reported to favour VEGFxxxb splice site selection in their human counterparts [14]. 3′UTR reverse primers designed to detect both pro- and anti-angiogenic VEGF families detected normal pro-angiogenic VEGFxxx products in both mouse and human cells and tissues, confirming that the PCR technique was effective. Qui *et al*., recently suggested that VEGFxxx isoforms may amplify preferentially in PCR reactions, even when VEGFxxx and VEGFxxxb transcripts are present at similar abundance. They proposed that differences in 3′UTR secondary structure between VEGFxxx and VEGFxxxb transcripts could cause differential reverse transcription efficiencies resulting in biased amplification in favour of VEGFxxx [15]. Whilst such a hypothesis could go some way towards explaining both our mouse and human data, it still does not adequately explain why we were so consistent in our inability to detect VEGFxxxb in human samples, since despite these potential structural issues, successful amplification of VEGFxxxb isoforms in the same tissues has been reported previously using this strategy [26], [28], [29], [39]. Moreover, no issues with cDNA synthesis strategies to accommodate potential structural issues have been discussed in previous studies reporting detection of VEGF165b, supporting the cDNA synthesis strategy adopted here. Interestingly, this approach did reveal a VEGF164/120 heteroduplex product migrating just below VEGF164 in wt solid fibrosarcomas and fibrosarcoma cells, as well as in all mouse tissues examined. Such a species has been reported previously in the literature and is thought to arise as a consequence of the PCR reaction itself [50]. Importantly, the heteroduplex species migrates in a manner analogous to VEGF145/144, a relatively rare VEGF isoform, highlighting the importance of sequencing all RT-PCR products in such studies, to be sure of their true identity. Heteroduplex formation could generally be an issue when considering PCR amplification of alternative splice variants. This is supported by the demonstration that heteroduplex species were generated when analysing isoforms of apoptosis regulators bcl-x [51] and caspase 10 [52].

Using a VEGFxxxb isoform-specific priming strategy, however, we did detect VEGFxxxb-like products in both mouse and human samples confirmed by DNA sequencing. However, the ability to detect these products was highly dependent on primer design, and was particularly dependent on the number of primer bases of complementary sequence spanning the exon8b/exon7 (for detection of VEGF164b/165b, 188b/189b products) or exon8b/exon5 splice junctions (for detection of VEGF120b/121b products). We believe this becomes important in analysing our data. Here, ability to detect VEGFxxxb products decreased as primer design became more discriminating towards VEGFxxxb amplification and this applied to both mouse and human cells and tissues. Thus, VEGFxxxb products were consistently amplified only when the reverse primer contained more bases of complementary sequence to exon7 or exon5 as opposed to exon8b. However, as discussed earlier, the VEGF188 and VEGF164 fibrosarcomas are inherently unable to splice the corresponding VEGFxxxb isoforms. It was also surprising that these VEGFxxxb products were generated at such high abundances (at least in wt and 188 tumours), since VEGFxxxb species are thought to be greatly down-regulated in tumours. We therefore propose that these products most likely represent misprimed VEGFxxx isoforms, amplified through annealing of the reverse primer to endogenous VEGFxxx transcripts. These transcripts exhibit an artefactual C-terminal exon 8b sequence as a consequence of exon 8b primer ‘tail’ amplification. This could explain why it became harder to detect putative VEGFxxxb products with primers exhibiting less sequence complementarity to exons 5 or 7, presumably through a decrease in probability for these primers to anneal to and successfully amplify endogenous VEGFxxx transcripts. Interestingly, we only detected VEGF164b with a reverse primer containing 5 as opposed to 4 bases complementary to exon 7, suggesting that this is the minimum number required to promote better annealing of the primer. However, this small amount of complementary sequence could mean that annealing is haphazard, accounting for the reproducibility issues we had with the detection of this species, especially at more stringent annealing temperatures. Analysis of the exon splice junction sequences in both the mouse and human VEGF-A genes reveals an identical sequence ^5′^TGCAG^3′^ present at the end of exons 3 and 4 as well as exon 7 (Fig. 8). On this basis, we hypothesise that any VEGFxxxb reverse primer designed to amplify VEGF164b/165b and VEGF188b/189b could in theory prime VEGFxxx and additional truncated products as a consequence of the PCR reaction. This is supported by detection of a 150 bp VEGF exon3/4/8b product in all mouse cDNAs, (Fig 6B & C). An intriguing possibility is that such truncated products could be primed from an existing bonafide VEGFxxxb transcript and appear more abundant simply because they are smaller and more readily amplified. However, the fact that the VEGF exon 3/4/8b species was readily detected in all fibrosarcomas including ones that could not splice into exon 8b makes this interpretation untenable. Our mispriming hypothesis is further strongly supported when considering RT-PCR data obtained using either cDNA extracts from VEGF164 and VEGF188 fibrosarcoma cells or plasmid DNAs corresponding to vectors used in the original construction of the VEF164 and VEGF188 expressing transgenic mice which cannot, due to their genetic construction splice VEGFxxxb isoforms. Here, the fact that all the VEGFxxxb isoform-specific primers that we employed generated plasmid-borne putative VEGFxxxb products misprimed from the VEGFxxx cDNAs inserted into the vectors, is most likely explained by the high abundance of purified VEGF cDNA available for the PCR reactions. Moreover, further support of our mispriming hypothesis is provided by the fact that both mouse and human VEGFxxxb isoform-specific primers spanning only 5 bases across the corresponding exon8b/7 junctions were able to amplify non-specifically, acetyl co-A and LIM domain only protein 7 isoform gene transcripts. We presume this occurred through mis-priming of primer through ^5′^GCAT^3′^ and ^5′^GCAG^3′^ sequences present in these two genes respectively. Whilst no studies have so far addressed VEGFxxxb isoform expression in the mouse, Artac *et al*., have recently reported the expression of VEGF188b and VEGF164b in the rat ovary [53]. This is intriguing considering that exon 8b is 100% conserved between rat and mouse. In their study, these authors used an isoform-specific real-time PCR approach but they did not report on any analyses using reverse primers capable of amplifying both the VEGFxxx and VEGFxxxb isoforms. Close examination of their primer design reveals that the reverse primer incorporated more complementary sequence to exon 7 (16 bases) than exon 8b (9 bases), a primer design, which in our study clearly yields VEGFxxx products exhibiting an artefactual exon 8b tail sequence.

Several studies have used the only commercially available anti-VEGFxxxb antibody (anti-human VEGF165b monoclonal antibody (clone 56–1 R&D Systems) for detecting VEGFxxxb isoforms at the protein level in rat and mouse tissues [43], [53], [54]. However, in our hands, this antibody unexpectedly detected protein(s) of equal size across our different isoform-specific mouse fibrosarcoma cell and solid tumour lines (Data S1). Furthermore, mass spectrometry of the 27 kDa protein band that was detected by the antibody in fibrosarcoma cell-conditioned media failed to identify VEGFxxxb proteins (data not shown). This puts into question the suitability of this antibody for analysing VEGFxxxb proteins in mouse and possibly rat which has a predicted 8b sequence (PLTGKTD) 100% homologous to that predicted for mouse.

In conclusion, we have presented compelling evidence that VEGFxxxb isoforms may not be commonly expressed in mice, and this might go some way to explaining the absence, as yet, of any VEGFxxxb transgenic mouse model. Interestingly, in support of our data, it is worth noting that the original *Vegfa^164/164^* and *Vegfa^188/188^* transgenic mice, that are incapable of expressing VEGFxxxb isoforms, did not exhibit hyper-vascular phenotypes, as might be expected if VEGFxxxb isoforms play an important role in counteracting the pro-angiogenic isoforms under normal circumstances. Indeed, the *Vegfa^164/164^* mice, in particular, appeared relatively normal [23], suggesting that any lack of anti-angiogenic isoforms has no major deleterious effects. Taken together, our data clearly highlight pitfalls associated with interpreting VEGF expression patterns. The general importance of RT-PCR primer design is highlighted, with a need for multiple approaches to discriminate isoform expression. RT-PCR using primers that can simultaneously amplify both VEGFxxx and VEGFxxxb species, we believe, provides the most reliable approach. Similarity between VEGFxxx/VEGFxxxb splice junction sequences in both mouse and human VEGF-A genes infers that the issues we highlight associated with interpreting VEGFxxxb isoform-specific RT-PCR mouse data can be extrapolated to include human data also, leaving us with the conundrum of why our experiments using human cDNAs failed to reveal authentic VEGFxxxb products. Consistent with our mouse data, the most obvious explanation is that VEGFxxxb products are not commonly expressed in the human cells and tissues we examined. Alternatively, they may be expressed at physiological levels below the limits of detection. Importantly, reports highlighting the anti-tumour effects of VEGF165b are based on *in vivo* studies using recombinant VEGF165b or overexpression of VEGF165b. Moreover, downstream signalling effects associated with endogenous VEGFxxxb isoforms remain, so far, unreported. Interestingly, Catena *et al*., recently report that VEGF121b and VEGF165b are weakly angiogenic rather than anti-angiogenic and upregulated rather than downregulated in breast cancer [55]. Consistent with our study, this data contradicts previous literature, and we believe adds strength to our hypothesis of xxxb primer ‘mispriming’ from existing VEGFxxx transcripts, since analysis of the primer design used in this study indicates VEGFxxxb isoform specific primers with significant homology to exons 5 (20 bases) or 7 (23 bases). These isoforms could therefore be VEGFxxx species with artefactual exon8b ‘tails’ which could therefore be weakly angiogenic compared to conventional VEGFxxx proteins, but would still be expected to be upregulated in cancer. Considering the potential importance associated with VEGFxxxb isofoms, our data, together with this data highlight the need for further clarification of many aspects of VEGFxxxb biology; the physiological existence of these isoforms, their expression levels and consequently their biological significance in keeping angiogenesis in check.

## Supporting Information

Data S1 **Provides supporting text showing methods, results and figure relating to characterisation of murine VEGF isoform expression by Western blotting and mass spectrometry.**
(DOC)
