# Supplementary material for: Do Anti-Angiogenic VEGF (VEGFxxxb) Isoforms Exist? A Cautionary Tale
Source: PLoS One. 2012 May 2;7(5):e35231. doi: 10.1371/journal.pone.0035231 (PMC3342274; doi:10.1371/journal.pone.0035231)
Supplement: Data S1 — Provides supporting text showing methods, results and figure relating to characterisation of murine VEGF isoform expression by Western blotting and mass spectrometry. (DOC) [file pone.0035231.s001.doc]

**Supplementary Data S1**
